# (p)ppGpp and c-di-AMP Homeostasis Is Controlled by CbpB in Listeria monocytogenes

**DOI:** 10.1128/mBio.01625-20

**Published:** 2020-08-25

**Authors:** Bret N. Peterson, Megan K. M. Young, Shukun Luo, Jeffrey Wang, Aaron T. Whiteley, Joshua J. Woodward, Liang Tong, Jue D. Wang, Daniel A. Portnoy

**Affiliations:** a Graduate Group in Microbiology, University of California, Berkeley, California, USA; b Department of Bacteriology, University of Wisconsin, Madison, Wisconsin, USA; c Department of Biological Sciences, Columbia University, New York, New York, USA; d Department of Molecular and Cell Biology, University of California, Berkeley, California, USA; e Graduate Group in Infectious Diseases and Immunity, University of California, Berkeley, California, USA; f Department of Microbiology, University of Washington, Seattle, Washington, USA; g Department of Plant and Microbial Biology, University of California, Berkeley, California, USA; University of Illinois at Chicago

**Keywords:** bacteria, cyclic dinucleotide, stringent response

## Abstract

The facultative intracellular pathogen Listeria monocytogenes, like many related *Firmicutes*, uses the nucleotide second messenger cyclic di-AMP (c-di-AMP) to adapt to changes in nutrient availability, osmotic stress, and the presence of cell wall-acting antibiotics. In rich medium, c-di-AMP is essential; however, mutations in *cbpB*, the gene encoding c-di-AMP binding protein B, suppress essentiality. In this study, we identified that the reason for *cbpB*-dependent essentiality is through induction of the stringent response by RelA. RelA is a bifunctional RelA/SpoT homolog (RSH) that modulates levels of (p)ppGpp, a secondary messenger that orchestrates the stringent response through multiple allosteric interactions. We performed a forward genetic suppressor screen on bacteria lacking c-di-AMP to identify genomic mutations that rescued growth while *cbpB* was constitutively expressed and identified mutations in the synthetase domain of RelA. The synthetase domain of RelA was also identified as an interacting partner of CbpB in a yeast-2-hybrid screen. Biochemical analyses confirmed that free CbpB activates RelA while c-di-AMP inhibits its activation. We solved the crystal structure of CbpB bound and unbound to c-di-AMP and provide insight into the region important for c-di-AMP binding and RelA activation. The results of this study show that CbpB completes a homeostatic regulatory circuit between c-di-AMP and (p)ppGpp in Listeria monocytogenes.

## INTRODUCTION

Nucleotide second messenger molecules are ubiquitous and widely conserved throughout all forms of life. The ever-expanding repertoire of nucleotide second messengers in bacteria highlights the complexity of their functions and interactions (1). Cyclic di-AMP (c-di-AMP) is a nucleotide second messenger that is highly conserved within Gram-positive bacteria, is present in *Archaea* and almost every bacterial phylum except *Gammaproteobacteria* (2), and is a member of the growing number of cyclic dinucleotides that include c-di-GMP, 3',3'-cGAMP, and 2',3'-cGAMP (3). c-di-AMP was discovered in the context of sporulation in Bacillus subtilis (4) but since then has been implicated in a wide variety of bacterial processes in nonsporulating bacteria, including maintenance of cell envelope integrity, osmoregulation, and central metabolism (5–9). During infection, c-di-AMP is secreted by Listeria monocytogenes into the host cell cytoplasm where it activates the host cyclic dinucleotide receptor STING, activating type I interferon signaling (10). Innate immune detection of this bacterial molecule implies a significant relationship between the two organisms throughout evolution and has prompted an in-depth analysis of the role of c-di-AMP in L. monocytogenes (11).

L. monocytogenes synthesizes c-di-AMP using a single diadenylate cyclase (DacA) and degrades it with two phosphodiesterases (PdeA and PgpH). Mutants that are unable to synthesize c-di-AMP are 10,000-fold less virulent in a mouse model of infection, are more susceptible to cell wall antibiotics, have increased growth rates in high solute concentrations, and are unable to grow in rich media unless supplemented with high salt concentrations (12–14). When c-di-AMP levels are elevated, as is the case when the genes encoding both c-di-AMP phosphodiesterases are deleted, bacteria are intolerant to high solute concentrations (14). Together, these observations highlight a relationship between c-di-AMP and regulation of cellular turgor pressure under various osmotic conditions. Accordingly, c-di-AMP binds to the carnitine transporter OpuCA and inhibits the uptake of organic osmolytes needed to respond to osmotic stress (7, 14). c-di-AMP also binds to the KtrCD potassium transporter complex, resulting in inhibition of potassium uptake, a process involved in osmotic stress response (15).

In rich media, c-di-AMP is essential for L. monocytogenes growth (5). This phenotype allowed for genetic approaches to understand signaling and identified suppressor mutations that restore the ability of c-di-AMP-deficient strains to grow in the lab. The most abundant mutations that circumvent the requirement for c-di-AMP in L. monocytogenes are in an oligopeptide permease (*opp*) operon. These findings identified that oligopeptides in rich medium are selectively toxic to bacteria in the absence of c-di-AMP. While disruption of Opp directly implicated peptides as a toxic element of rich media and suggested a role for c-di-AMP in osmoregulation (16, 17), it did not provide a molecular mechanism that explained why c-di-AMP is essential. Other suppressor mutations were found in pyruvate carboxylase (*pycA*), citrate synthase (*citZ*), c-di-AMP binding protein-encoding genes *pstA* and *cbpB*, and a bifunctional (p)ppGpp synthetase/hydrolase (*relA*). Mutations in *pycA*, *citZ*, and *pstA* suppressed not only essentiality of c-di-AMP but also the sensitivity of that strain to β-lactam antibiotics, suggesting these mutations may all affect the same c-di-AMP-regulated metabolic pathway, distinct from *relA* and *cbpB*.

Analysis of suppressor mutations in *relA* led to the observation that L. monocytogenes mutants lacking c-di-AMP accumulate guanosine tetra- and pentaphosphate [ppGpp and pppGpp, collectively referred to as (p)ppGpp], which are selectively toxic due to downstream transcriptional changes involving the *codY* regulon (5). (p)ppGpp are linear nucleotide second messengers that direct the reorganization of cellular processes away from growth and toward synthesis of genes involved in cell survival and metabolite biosynthesis (18). This process is termed the “stringent response” (19). The best-characterized stimulus for induction of the stringent response is uncharged tRNAs that accumulate during amino acid starvation and cause actively translating ribosomes to stall, activating the synthetase domain of RelA (20). In Gram-positive bacteria, RelA (also known as RelA/SpoT homolog [RSH]) is a bifunctional (p)ppGpp synthetase/hydrolase that adopts opposing conformations to regulate two distinct catalytic activities (20). The balance of RelA conformations dictates (p)ppGpp levels. RSH enzymes are highly conserved across bacteria and archaea and even in plant chloroplasts and induce a common general starvation and stress response that spans microbial life (21).

It is unclear why depletion of c-di-AMP in L. monocytogenes leads to accumulation of (p)ppGpp under nonstarvation conditions, and here, we explored the molecular mechanism that underlies this nucleotide second messenger cross talk. Alternative stimuli for (p)ppGpp synthesis have been identified in related organisms, including transcriptional induction of small alarmone synthetases RelP and RelQ, and other starvation conditions (21–25). However, here we found that CbpB is a direct activator of RelA and that c-di-AMP inhibits CbpB-dependent RelA activation. *In vivo* CbpB was responsible for elevating cellular concentrations of (p)ppGpp in response to reduced c-di-AMP levels by regulating the enzymatic functions of RelA. Our model indicates that homeostasis of c-di-AMP and (p)ppGpp is maintained through enzymatic regulation of RelA, c-di-AMP phosphodiesterases, and nucleotide cross talk.

## RESULTS

### CbpB is toxic to bacteria deficient for c-di-AMP.

**FIG 1:**
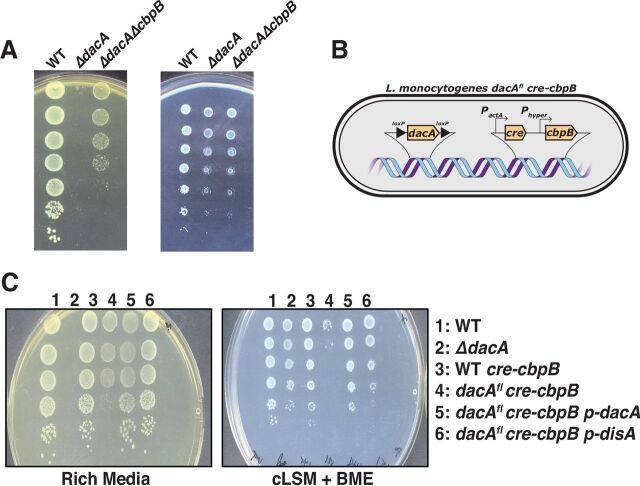
CbpB is toxic in the absence of c-di-AMP. (A) *dacA* deletion mutants are unable to grow on rich media unless there is a second mutation in *cbpB* that renders it nonfunctional. All three strains grow on complete listeria synthetic medium (cLSM). (B) Construction of a strain to test CbpB function. The *dacA^fl^ cre-cbpB* strain was engineered to flox the *dacA* gene while constitutively expressing *cbpB*. (C) When plating this strain on *actA-*activating medium, the resulting mutant is Δ*dacA P_hyper_-cbpB.* Plating this strain on *actA*-activating medium also resulted in a plating deficiency, which was restored by the addition of L. monocytogenes *dacA* or B. subtilis *disA*.

We previously reported that *dacA* is essential for bacterial growth under conventional laboratory conditions (5). In rich medium, *ΔdacA* mutants are recovered only if they harbor suppressor mutations that fall into several broad categories: osmolyte uptake (*oppABCDF* and *gbuABC*), central metabolism (*pycA* and *pstA*) (6, 13), the stringent response (*relA*), and a gene of unknown function that encodes cyclic di-AMP binding protein B (*cbpB*). The molecular mechanisms linking c-di-AMP signaling to broad phenotypes are an area of active investigation, and here, we selected the c-di-AMP binding protein CbpB for further analysis. Strains of L. monocytogenes that are unable to synthesize c-di-AMP could not grow on rich medium unless *cbpB* was also inactivated (Fig. 1A). Mutations in *dacA* and/or *cbpB* were constructed in bacteria grown in synthetic medium, and then mutants were plated on either synthetic or rich medium. L. monocytogenes Δ*dacA* mutants exhibit a >4-log plating defect on rich medium while *ΔdacA ΔcbpB* mutants were defective by only ∼1 log.

*cbpB* encodes a 150-amino-acid protein that is conserved in some *Firmicutes*. The CbpB protein is predicted to adopt two cystathionine beta synthase (CBS) domains and was previously identified in an unbiased screen for c-di-AMP binding proteins (6). CbpB mutations had no effect on growth of wild-type (WT) L. monocytogenes in a mouse model of virulence (see Fig. S1 in the supplemental material). Similarly, overexpression of *cbpB* in wild-type bacteria did not produce a phenotype in a mouse model of infection (Fig. S1). However, overexpressing *cbpB* in bacteria lacking c-di-AMP appeared toxic even in synthetic medium (Fig. 1C). This mutant was made by constructing a mutant that allowed for inducible deletion of *dacA* by flanking chromosomal *dacA* with *loxP* sites as previously described (5) and then expressing *cre* using an inducible promoter (Fig. 1B) (see Materials and Methods for full details). In this experiment, medium-inducible deletion of *dacA* in strains overexpressing *cbpB* led to a plating deficiency of ∼10^−4^ compared to WT; however, expression of *dacA* or an unrelated c-di-AMP synthase from B. subtilis, *disA*, restored the plating efficiency to wild-type levels. These data suggested that c-di-AMP counteracted the toxicity of CbpB, either by directly binding to and inactivating the protein or through altering the physiology of L. monocytogenes.

10.1128/mBio.01625-20.1 FIG S1 CbpB does not affect the virulence of L. monocytogenes. Virulence of L. monocytogenes in spleens and livers of CD-1 mice. Horizontal lines represent medians. Download FIG S1, TIF file, 2.0 MB. Copyright © 2020 Peterson et al. 2020 Peterson et al. This content is distributed under the terms of the Creative Commons Attribution 4.0 International license.

### RelA activation is a function of CbpB *in vivo*.

**FIG 2:**
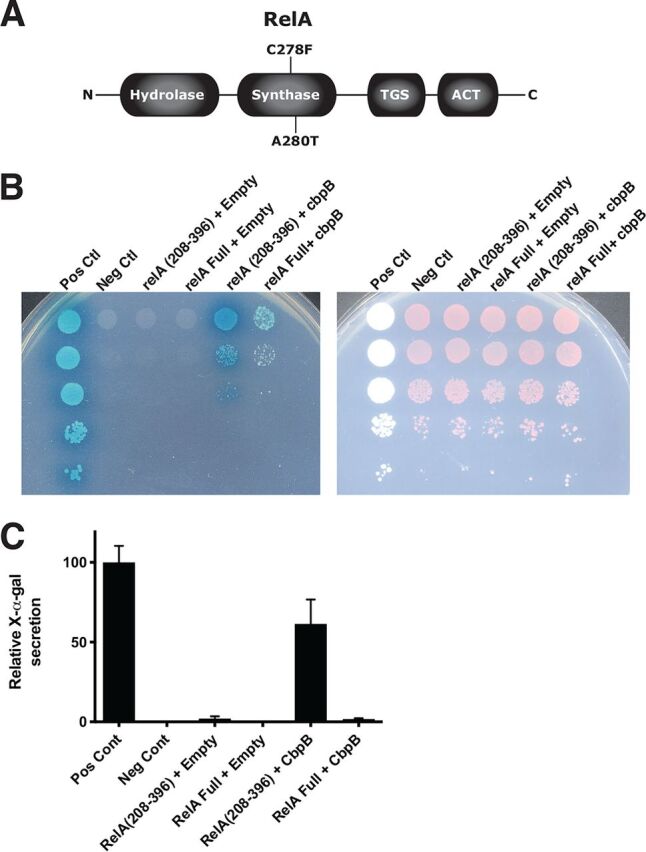
CbpB directly activates the synthetase domain of RelA. (A) RelA was the predominant mutation identified through sequencing of suppressor Δ*dacA cre-cbpB* strains. (B) Confirmation of yeast-2-hybrid results showed that RelA can interact with CbpB. (Left) QDO/X/A medium allows for growth only when subunits are interacting. (Right) DDO medium allows for growth when both prey and bait plasmids are present. See Materials and Methods. (C) Quantification of X-α-gal activity. Bar heights represent averages from three independent experiments with error bars representing the standard deviation.

CbpB binds c-di-AMP, leading us to hypothesize that nucleotide binding disrupts a protein-protein interaction that is selectively toxic to *dacA* mutants. We undertook two parallel approaches to identify CbpB binding partners and reasoned that the combination of unrelated methods should rapidly eliminate false-positive findings. We first took a genetic approach to understanding CbpB by isolating suppressor mutations. Previously, our lab has isolated suppressor mutations that allow Δ*dacA* strains to grow in rich medium. In this analysis, we focused specifically on mutations that suppress the function of CbpB by utilizing *cbpB* overexpression. L. monocytogenes *ΔdacA* strains overexpressing *cbpB* were recovered at a reduced frequency (∼10^−4^) in synthetic medium (Fig. 1C); however, colonies of bacteria could still be recovered, suggesting these strains may harbor suppressor mutations that specifically bypass CbpB toxicity. Genome sequencing of 25 independently isolated colonies revealed that each strain had at least one mutation (Table 1). The majority of mutations were located within the *cbpB* gene and presumably inactivated its function. Interestingly, the next most frequent mutations were in genes related to (p)ppGpp signaling, either in the synthetase domain of the *relA* gene (Fig. 2A), encoding a bifunctional (p)ppGpp synthetase/hydrolase, or in *hflX*, encoding a major (p)ppGpp receptor (26). This suggested a connection between CbpB and the alarmones (p)ppGpp. Other mutated genes in these strains were identified only once and did not have an obvious connection with (p)ppGpp (Table 1).

**TABLE 1:**
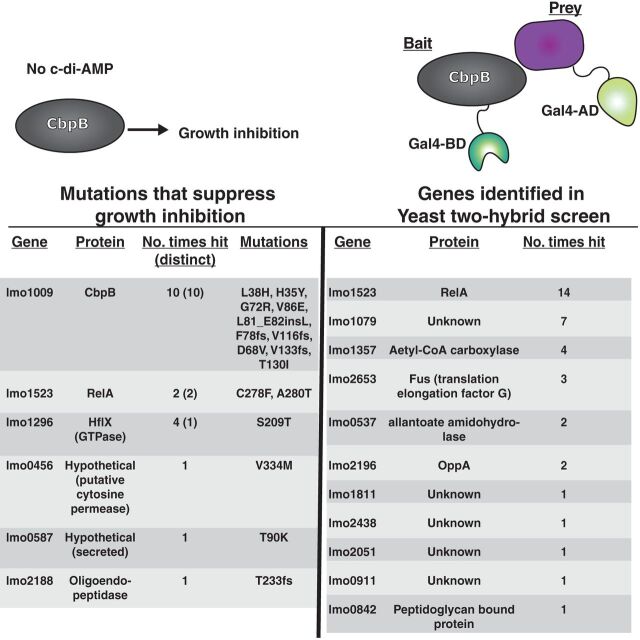
Parallel screens identify genes involved in *cbpB* function^*a*^

a Left side: genomic mutations that allow for growth of Δ*dacA P_hyper_-cbpB.* Right side: protein coding genes that were identified as CbpB interactors by yeast-2-hybrid assay.

The second approach we took to identify CbpB binding partners was an unbiased yeast-2-hybrid analysis, using CbpB as a bait and a genomic library of L. monocytogenes as prey. From an estimated 100,000 independent prey constructs, we identified 11 candidate loci exhibiting a positive yeast-2-hybrid interaction (Table 1). The most frequently isolated locus that appeared to positively interact with the *cbpB* bait was *relA*. Interestingly, the *relA*-carrying prey plasmid identified expresses only a fragment of RelA (amino acids 208 to 396), which maps to the synthetase region of the protein. We confirmed the yeast-2-hybrid results by pairing empty or *cbpB*-carrying bait plasmids with full-length or truncated *relA*-carrying prey plasmids (Fig. 2B and C). Interactions between *relA* and *cbpB* appeared to be specific for the *cbpB* bait and strongest for the isolated *relA* fragment.

### CbpB activates the (p)ppGpp synthetase activity of RelA.

**FIG 3:**
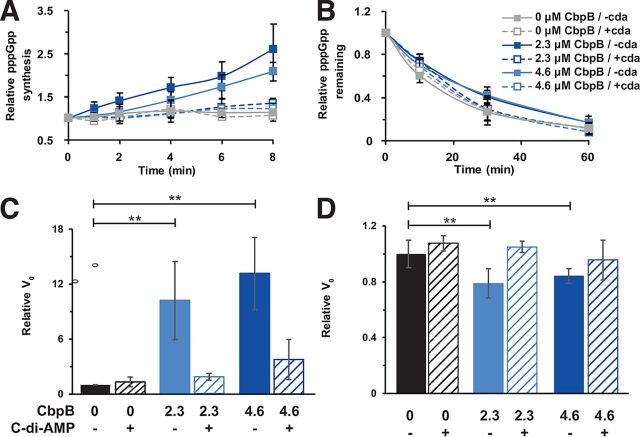
CbpB activates the synthesis of (p)ppGpp by RelA. RelA synthetase and hydrolase activities were assayed *in vitro* to test the effects of CbpB and c-di-AMP. RelA concentration was held constant at 1.5 μM, while CbpB and c-di-AMP were added at 2.3 μM or 4.6 μM. (A) RelA synthetase activity was tested by observing formation of [γ-^32^P]pppGpp from [γ-^32^P]GTP and ATP. (B) RelA hydrolase activity was tested by observing hydrolysis of [γ-^32^P]pppGpp into [γ-^32^P]GTP. Three replicates were performed for each reaction. (C) As evidenced by the respective *V*_0_ values, RelA synthetase activity was strongly enhanced in the presence of either concentration of CbpB tested, but the effect diminished with the addition of c-di-AMP. (D) Conversely, RelA hydrolase activity was slightly reduced in the presence of either concentration of CbpB, and addition of c-di-AMP reverted to control levels. *V*_0_ values are in relative radioactivity units. *P* values to assess the effects of CbpB, cyclic-di-AMP, and their interaction were calculated by two-way analysis of covariance test. V_0_, initial velocity. **, *P* ≤ 0.01.

Genetic evidence from suppressor mutations and the yeast-2-hybrid screen suggested that CbpB may interact with RelA. RelA is a bifunctional synthetase/hydrolase of the linear nucleotide second messenger (p)ppGpp. RelA associates with the ribosome and monitors the presence of uncharged tRNA molecules during translation as a surrogate for amino acid starvation. While (p)ppGpp synthesis is also mediated through RelP and RelQ (27), and there is evidence for an additional hydrolase gene (21), RelA is the only confirmed (p)ppGpp hydrolase in L. monocytogenes. We reasoned that CbpB might alter (p)ppGpp levels by stimulating or inhibiting an enzymatic function of RelA. L. monocytogenes RelA and CbpB were expressed recombinantly from Escherichia coli, and we monitored the activity of RelA to synthesize pppGpp *in vitro.* While RelA is unable to synthesize pppGpp on its own, addition of CbpB to RelA resulted in dramatic activation of pppGpp synthesis compared to RelA alone (Fig. 3A). Furthermore, addition of c-di-AMP ablated the CbpB-dependent RelA reaction but had no effect on RelA alone. Similarly, CbpB inhibited pppGpp hydrolysis by RelA, albeit to a modest extent (Fig. 3B). Accordingly, the initial velocity of pppGpp synthesis was higher in the presence of a 2:1 and 4:1 ratio of CbpB to RelA (Fig. 3C). However, initial velocities of pppGpp hydrolysis were mildly affected by the presence of CbpB (Fig. 3D). The yeast-2-hybrid data demonstrated that the synthetase domain of RelA is sufficient to mediate CbpB-RelA interactions. Taken together, these data suggested a model where CbpB interacts with the RelA synthetase domain and leads to activation of (p)ppGpp production that is independent of the ribosome or starvation.

### Structural insights into the binding of CbpB by c-di-AMP.

**FIG 4:**
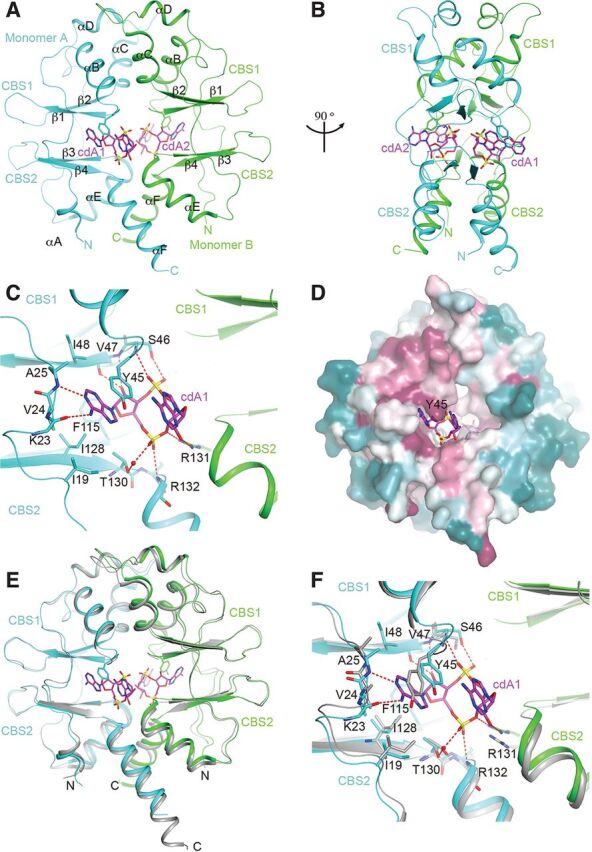
Crystal structure of CbpB in complex with c-di-AMP reveals a mode of inhibition. (A) Overall structure of the CbpB homodimer (monomer A in cyan, monomer B in green) in complex with c-di-AMP (labeled cdA1 and cdA2; carbon atoms colored magenta, nitrogens colored blue, phosphorus colored yellow, and oxygen colored red). The two tandem CBS domains in each monomer are labeled CBS1 and CBS2. Helices and strands in the two monomers are also labeled. (B) Overall structure of the CbpB homodimer in complex with c-di-AMP, after a 90° rotation around the vertical axis. (C) Detailed interactions between CbpB and c-di-AMP. Hydrogen bonds and water molecule are shown with red dashed lines and a red sphere. Residues involved in interaction are also labeled. The side chains of Arg132 and Lys32 are not shown for clarity. (D) Sequence conservation of CbpB homologs, generated by the program ConSurf (43) based on an alignment of 150 sequences selected automatically. Purple indicates conserved residues, cyan indicates variable residues, and white indicates average conservation. (E) Overlay of the structure of the CbpB homodimer (monomer A in cyan, monomer B in green) in complex with c-di-AMP (magenta) with that of free CbpB (gray). (F) Structural differences in the binding site for c-di-AMP in free CbpB. The side chains of Arg132 and Lys32 are not shown for clarity.

The biochemical data described above suggested that CbpB is inactivated by c-di-AMP, and we next sought to determine the molecular mechanism for this interaction. The CbpB protein consists of two CBS domains and was previously shown to bind c-di-AMP with high affinity (6). We determined the structures of free CbpB and CbpB in complex with c-di-AMP at 1.6- and 2.4-Å resolution, respectively (Table S1). These structures reveal that CbpB has tandem CBS domains (CBS1 and CBS2) that interact to form a disk-like head-to-head homodimer. In the c-di-AMP complex, one U-shaped c-di-AMP molecule is bound to each face of the disk (Fig. 4A and B), and the binding modes of the two c-di-AMP molecules are essentially the same. One of the adenine bases of c-di-AMP is located in the cleft between the two tandem CBS domains (CBS1 and CBS2) of each monomer, while the other is projected into the solvent.

10.1128/mBio.01625-20.6 TABLE S1 Summary of data collection and refinement statistics. Download Table S1, TIF file, 2.0 MB. Copyright © 2020 Peterson et al. 2020 Peterson et al. This content is distributed under the terms of the Creative Commons Attribution 4.0 International license.

Specific recognition of c-di-AMP is achieved by a large number of hydrogen bonds with both main chains and side chains of CbpB, some of which are mediated by water molecules. The adenine base in the cleft is recognized through hydrogen bonds to its N-1 and N-6 atoms (Fig. 4C). It is π-stacked against Tyr45 on one face while its other face is flanked by Ile19 and Ile128, thereby forming a narrow pocket for the binding of this base. Its ribose is positioned against the side chain of Phe115, and the 2' hydroxyl group has hydrogen-bonding interactions with the main chain of Val47. The 5' phosphate is recognized by hydrogen-bonding interactions with the main chain amide of Arg132, ionic interactions with Arg131 from the other monomer, and hydrogen-bonding interactions with the side chain hydroxyl of Thr130 through a water molecule. This phosphate is situated in a generally positively charged region of the structure.

In contrast, the other adenine base of c-di-AMP has no hydrogen-bonding interactions with CbpB, and it is not flanked by residues from the protein either, except that its N-6 atom is positioned against Tyr45 (Fig. 4C). The 2' hydroxyl group of its ribose has hydrogen-bonding interactions with the side chain of Arg131 from the other monomer. The 5' phosphate has hydrogen-bonding interactions with the main chain and side chain of Ser46. One of the terminal oxygen atoms on this phosphate is 4.4 Å away from the equivalent atom in the c-di-AMP molecule on the other face of the disk (Fig. 4B).

This binding site of c-di-AMP, especially Tyr45 and the residues flanking the adenine on the other face, is well conserved among CbpB homologs (Fig. 4D). This analysis reveals another conserved surface patch, located away from the c-di-AMP binding site and formed by residues in CBS1, which may mediate a separate function of this protein.

The binding mode of c-di-AMP to CbpB differs from that of OpuC, another CBS-containing c-di-AMP binding protein. In this case, c-di-AMP assumes an extended conformation in the complex with OpuC (14), such that the two bases are recognized equivalently. In addition, the rings of c-di-AMP are flipped ∼180° in the two complexes, such that the adenine bases are positioned deeper into the protein in the OpuC complex. Overall, the binding mode of c-di-AMP in CbpB defines a new type of nucleotide recognition by CBS domains.

The structure of free CbpB is similar to the c-di-AMP complex, with root mean square (RMS) distance of 0.86 Å between their equivalent Cα atoms (Fig. 4E). The major differences between the two structures are within the c-di-AMP binding site. Notably, the conformation of the Tyr45 side chain in free CbpB has severe clashes with the bound position of c-di-AMP (Fig. 4F), and therefore the binding site does not exist in free CbpB. While these structural differences exist, they do not provide obvious explanations for how CbpB-RelA interactions might be regulated.

An analysis of the single-amino-acid mutations of CbpB that allowed for growth in the absence of c-di-AMP reveals three categories of suppressor mutants (Fig. S2). The first category includes T130I, a residue involved in c-di-AMP binding (Fig. 4C). The second category includes V86E and is in the hydrophobic core of CBS1. The side chain of Glu will likely destabilize this domain and possibly the entire protein. The third category maps to the conserved surface of CbpB, which is far away from the c-di-AMP binding site and could be involved in another function (Fig. 4D). These mutations are H35Y, L38H, D68V, and G72R. Although this analysis is not conclusive, it suggests that this region could be important for the interaction with RelA.

10.1128/mBio.01625-20.2 FIG S2 Structural analysis of CbpB mutations that suppress growth inhibition. T130 is within the c-di-AMP binding region and is probably important for complex formation. V86 is in the hydrophobic core of CBS1. H35, L38, D68, and G72 are probably important for additional functions due to their distance from the c-di-AMP binding region. Download FIG S2, TIF file, 2.2 MB. Copyright © 2020 Peterson et al. 2020 Peterson et al. This content is distributed under the terms of the Creative Commons Attribution 4.0 International license.

### CbpB increases (p)ppGpp levels *in vivo*, linking c-di-AMP with (p)ppGpp signaling.

**FIG 5:**
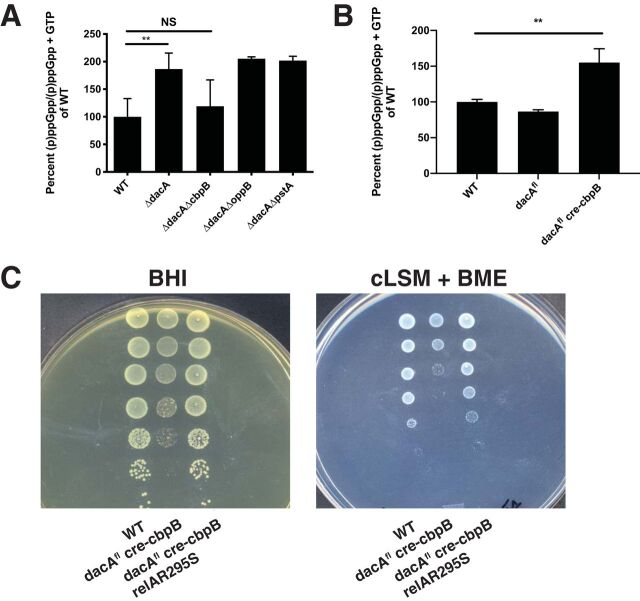
CbpB toxicity is mediated by the synthesis of (p)ppGpp by RelA. (A) Deletion in *cbpB* restores Δ*dacA* (p)ppGpp levels to WT level as measured by TLC. Deletions in *pstA* and *oppB* (which also suppress *dacA* essentiality) do not affect alarmone levels, indicating alternative pathways of maintaining c-di-AMP essentiality. (B) CbpB induces the stringent response under low-c-di-AMP conditions. Importantly, these strains were not grown under conditions that induce expression of the *cre* recombinase. (C) Transduction of a synthetase-dead mutant into the *dacA^fl^ cre-cbpB* mutant relieves CbpB-mediated toxicity when *dacA* is deleted. Bar heights represent averages from three independent experiments with error bars representing the standard deviation. NS, not significant; **, significant by unpaired *t* test (*P* ≤ 0.01).

The *dacA* gene is essential for growth of L. monocytogenes in rich medium partly due to accumulation of (p)ppGpp (5). The molecular mechanism explaining this phenomenon is not immediately clear because rich medium should not induce a starvation response. We directly measured accumulation of (p)ppGpp in L. monocytogenes strains lacking c-di-AMP and compared the effects of deleting *cbpB*. The *ΔdacA* strain exhibited increased (p)ppGpp {measured as a ratio of (p)ppGpp/[(p)ppGpp + GTP]} while (p)ppGpp levels in the *ΔdacA ΔcbpB* strain are comparable to wild type (Fig. 5A). Mutations in *cbpB* were uniquely able to ameliorate increased (p)ppGpp among other previously isolated suppressor mutations. Consistent with the role of CbpB activating RelA in the absence of c-di-AMP, overexpression of *cbpB* in a *dacA* knockdown strain (see figure legends) produced higher levels of (p)ppGpp than WT and *dacA* knockdown strains (Fig. 5B). Although *dacA* had not been deleted under these conditions, we know it produces lower levels of the c-di-AMP due to the placement of the *loxP* site 5' to the *dacA* open reading frame (ORF) (Fig. S3) (see Materials and Methods).

10.1128/mBio.01625-20.3 FIG S3 loxP sites affect *dacA* expression. Western blot of WT, *loxP-dacA-loxP*, and Δ*dacA* lysates. Download FIG S3, TIF file, 2.0 MB. Copyright © 2020 Peterson et al. 2020 Peterson et al. This content is distributed under the terms of the Creative Commons Attribution 4.0 International license.

These data suggest that overexpression of *cbpB* is toxic in a *ΔdacA* mutant background due to overproduction of (p)ppGpp by RelA. We tested this hypothesis by overexpressing *cbpB* in a *ΔdacA* mutant strain unable to synthesize (p)ppGpp through RelA (RelA synthetase-dead mutant allele R295S) (5). In line with our predictions, CbpB expression no longer inhibited growth, indicating that synthesis of (p)ppGpp by RelA is responsible for CbpB toxicity (Fig. 5C).

## DISCUSSION

c-di-AMP is unlike many other nucleotide second messengers because it is essential and constitutively produced under almost all growth conditions yet analyzed. In this study, we investigated one aspect of c-di-AMP essentiality in L. monocytogenes. In L. monocytogenes, c-di-AMP binds to the proteins PycA, PstA, OpuC, KdpD, KtrC, CbpA, and CbpB (6, 7, 14, 15). The catalytic activity of PycA and transporter activation by OpuC and KtrC are inhibited upon nucleotide binding; however, the function of other c-di-AMP binding proteins such as CbpB is poorly understood. Mutations in CbpB suppressed the essentiality of c-di-AMP, and expression of CbpB was selectively toxic in the absence of c-di-AMP. We used multiple unbiased screens to identify a protein-protein interaction between CbpB and RelA, a bifunctional synthetase/hydrolase of the stringent response mediator (p)ppGpp. *In vitro* analysis confirmed that CbpB activates (p)ppGpp synthesis by interacting with the RelA synthetase domain. L. monocytogenes strains lacking c-di-AMP cannot grow due to elevated levels of (p)ppGpp; however, additional mutation of *cbpB* returned (p)ppGpp to wild-type levels. These data lead to a model of direct, cross-nucleotide signaling, between c-di-AMP and the (p)ppGpp that is mediated by CbpB.

The crystal structure of CbpB confirms the presence of the two predicted CBS domains and that these domains mediate c-di-AMP binding. CBS domains are found in two other c-di-AMP-interacting proteins encoded by L. monocytogenes, CbpA and OpuCA. Crystal structures of OpuC are similar to CbpB, except the bound c-di-AMP bridges across the two OpuC monomers whereas CbpB monomers bind individual c-di-AMP molecules. The CbpB structure exhibits very little change upon c-di-AMP binding and appears as a homodimer in both the free and nucleotide-bound forms, similar to the crystal structure of the CbpB homolog from B. subtilis, DarB (PDB1YAV). It remains unclear how such modest structural changes result in such large impacts on protein-protein interactions, and future work should compare these structural characteristics with those of a CbpB-RelA complex.

**FIG 6:**
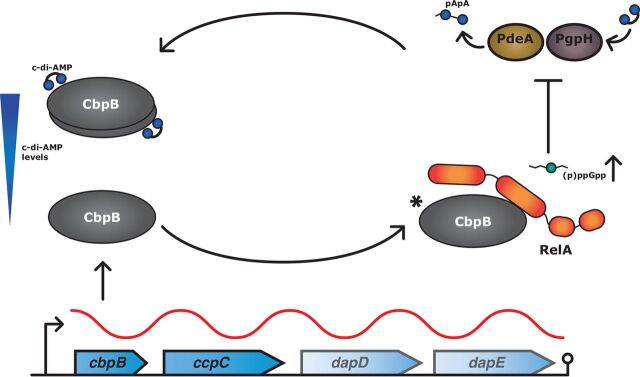
A model for c-di-AMP and (p)ppGpp homeostasis. As c-di-AMP levels drop and *cbpB* is expressed, CbpB becomes free to bind RelA and activate its (p)ppGpp synthetase domain. Increased (p)ppGpp results in inhibition of phosphodiesterases PdeA and PgpH, leading to a buildup in c-di-AMP. c-di-AMP binds to free CbpB and prevents stringent activation. The asterisk indicates that the molar ratio of CbpB to RelA is unknown.

The level of c-di-AMP within the cells of *Firmicutes* is maintained by balancing the activity of diadenylate cyclases and phosphodiesterases that synthesize and degrade c-di-AMP, respectively. Interestingly, (p)ppGpp inhibits both known c-di-AMP-specific phosphodiesterases in L. monocytogenes, creating a feedback circuit between these two signaling molecules (28–30). The phosphodiesterases PdeA (a DHH-DHHA1 domain-containing enzyme homologous to GdpP) and PgpH (an HD-domain-containing enzyme) are mechanistically distinct, suggesting that (p)ppGpp-dependent regulation of these enzymes evolved independently. Our work demonstrates that as c-di-AMP decreases, CbpB activates RelA and leads to (p)ppGpp synthesis, which in turn would inhibit c-di-AMP hydrolysis and return c-di-AMP levels to normal (Fig. 6). Thus, CbpB and PdeA/PgpH (c-di-AMP phosphodiesterases) form a homeostatic signaling loop to maintain appropriate c-di-AMP levels within the cell.

The stringent response in *Firmicutes* allows for adaptation to multiple cell stressors including starvation and cell envelope stress (31); work presented here helps to uncover how diverse inputs can induce the stringent response. The best-understood mechanism for stringent response induction is the activation of RelA by uncharged tRNA molecules encountered at the ribosome during translation. We were intrigued by the possibility that the CbpB-RelA interaction might localize to the ribosome; however, multiple attempts to detect CbpB within polysomal fractions of Δ*dacA* mutants indicated CbpB only within light-weight fractions (see Fig. S4 in the supplemental material). Our work establishes that CbpB can stimulate RelA to synthesize (p)ppGpp in a ribosome-independent manner and adds to a growing list of noncanonical stimuli for RelA regulation. In E. coli, RSD and acyl-carrier protein bind SpoT (a RelA homolog) during carbon and lipid starvation, respectively, and induce opposing hydrolase or synthetase activities (22, 24). In Caulobacter crescentus, EII^NTR^∼P can bind SpoT in response to nitrogen starvation and inhibits the hydrolase domain (32). And in multiple bacterial phyla, branched-chain amino acids bind to RelA and regulate (p)ppGpp hydrolysis (23). These mechanisms offer a direct link between a variety of starvation conditions (carbon, nitrogen, fatty acid, phosphate, and iron), as well as cell envelope stress, and stringent response induction through noncanonical inputs to (p)ppGpp synthesis.

10.1128/mBio.01625-20.4 FIG S4 Polysome profile of Δ*dacA P_hyper_-cbpB.* Whole-cell lysates (WCL) and polysomal fractions were harvested from bacteria and analyzed for the presence of CbpB by Western blotting. Only the WCL and lightweight fractions contained CbpB. The higher-weight fractions did not indicate the presence of CbpB. Download FIG S4, TIF file, 2.0 MB. Copyright © 2020 Peterson et al. 2020 Peterson et al. This content is distributed under the terms of the Creative Commons Attribution 4.0 International license.

At least two conditions must be satisfied for CbpB to activate RelA: c-di-AMP levels must be low enough to no longer inactivate CbpB and *cbpB* must be transcribed and translated (Fig. 6). The genomic context of *cbpB* is conserved between L. monocytogenes and B. subtilis and provides a clue as to when it is expressed. *cbpB* is found in an operon with the transcriptional regulator, *ccpC* (Fig. 6). CcpC represses transcription of genes encoding the oxidative branch of the tricarboxylic acid (TCA) cycle, but CcpC is inhibited when bound to citrate. When citrate levels increase, CcpC remodels transcription to increase carbon flow through the TCA cycle and deplete excess citrate, as well as derepressing its own operon (33–36). The relationship of citrate levels to *cbpB* expression appears enigmatic yet pertinent because management of citrate concentrations in the cell is of particular concern for L. monocytogenes Δ*dacA* mutants, to which citrate appears selectively toxic (13). CbpB is not universally conserved and does not appear to be encoded by Staphylococcus aureus. This observation is in line with other evidence for a different relationship between c-di-AMP and (p)ppGpp in that organism. While depletion of c-di-AMP leads to stringent response activations in L. monocytogenes, overproduction of c-di-AMP induces the same response in S. aureus (37). The molecular mechanism for how increased c-di-AMP increases (p)ppGpp in S. aureus is yet to be elucidated, but it will be interesting to see if there are c-di-AMP-responsive factors that mediate cross talk with (p)ppGpp.

The unifying function of c-di-AMP is alteration of internal osmotic pressure, and c-di-AMP-dependent osmoregulation is essential for growth of L. monocytogenes in rich medium. Either addition of salt, removal of nutritive oligopeptides, or additional suppressor mutations can circumvent the requirement of L. monocytogenes for the *dacA* gene. Analysis of Δ*dacA* suppressor mutations and c-di-AMP binding proteins suggests a model where increased intracellular citrate, nutritive oligopeptides, and high levels of (p)ppGpp combine to inhibit growth in the absence of c-di-AMP (5, 13). Citrate accumulates in Δ*dacA* mutants because in wild-type bacteria, c-di-AMP regulates carbon flow through the oxidative branch of the TCA cycle by binding and inhibiting pyruvate carboxylase (PycA) and by binding PstA (6, 13). Increased intracellular citrate also sensitizes Δ*dacA* mutants to beta-lactam antibiotics (12, 13). CbpB did not affect citrate production in Δ*dacA* mutants, and consistently, *cbpB* mutations do not alter sensitivity to beta-lactam antibiotics (13) (Fig. S5). (p)ppGpp accumulates in Δ*dacA* mutants and is interrupted only by mutations in *relA*, the primary (p)ppGpp synthetase, and *cbpB*, which activates RelA. The toxicity of (p)ppGpp in Δ*dacA* is a consequence of derepression of the CodY regulon; however, these transcriptional changes are toxic only when combined with increased citrate and nutritive oligopeptides. It remains to be determined if these three corollaries independently contribute to osmoregulation in an additive manner or if instead they converge/synergize into one central driver of essentiality.

10.1128/mBio.01625-20.5 FIG S5 Citrate production from WT and Δ*dacA* variants. Production of citrate as a proxy for carbon flux from WT and Δ*dacA* strains and Δ*dacA* mutants containing suppressor mutations that allow for growth on rich media. The buildup of citrate in the Δ*dacA* Δ*cbpB* strain suggests that citrate production *per se* is not a toxic metabolite to this mutant. Bars represent averages from three independent experiments with error bars representing the standard error. Download FIG S5, TIF file, 2.0 MB. Copyright © 2020 Peterson et al. 2020 Peterson et al. This content is distributed under the terms of the Creative Commons Attribution 4.0 International license.

The work mechanistically answers a standing question of why (p)ppGpp accumulates in c-di-AMP-deficient bacteria even in rich medium by linking a c-di-AMP binding protein to a (p)ppGpp synthetase. Manipulation of c-di-AMP levels has demonstrated how critical this second messenger is to growth and cellular viability, resistance to cell wall-acting antibiotics, osmolyte/ion transport across the membrane, and metabolism. The next outstanding question for the field is determination of the physiological inputs that alter cellular c-di-AMP levels in nature and how these mechanisms allow for diverse microbes to grow in their respective replicative niches.

## MATERIALS AND METHODS

### Ethics statement.

This study was carried out in strict accordance with the recommendations in the *Guide for the Care and Use of Laboratory Animals* of the National Research Council of the National Academy of Sciences (45). All protocols were reviewed and approved by the Animal Care and Use Committee at the University of California, Berkeley (AUP-2016-05-8811).

### Bacterial culture conditions.

The L. monocytogenes strains used in this study (see Table S2 in the supplemental material) (44) were derived from wild-type 10403S. They were maintained in brain heart infusion (BHI; Difco) for *dacA*^+^ strains and on listeria synthetic medium (LSM) for *dacA* mutant strains, in 1 to 3 ml of medium in 14-ml (17- by 100-mm) culture tubes, at 37°C while shaking at 220 rpm, unless specified otherwise.

10.1128/mBio.01625-20.7 TABLE S2 L. monocytogenes strains used in this study. Download Table S2, TIF file, 2.5 MB. Copyright © 2020 Peterson et al. 2020 Peterson et al. This content is distributed under the terms of the Creative Commons Attribution 4.0 International license.

### Listeria synthetic medium.

The LSM was made using a previously described recipe (13). LSM was prepared by combining eight individual concentrated stock solutions (prepared ahead of time and stored at 4°C) with fresh glutamine and cysteine (see Table S1). Stock solutions were filter sterilized and stored at 4°C, with the exception of morpholinepropanesulfonic acid (MOPS), glucose, and phosphate, which were stored at room temperature. Each stock was added at the appropriate dilution factor and in the order listed in Table S1, dissolving glutamine and cysteine immediately prior to filter sterilization. The final LSM is stable at a 2× concentration and for approximately 6 to 8 weeks. LSM‐agar plates are prepared by combining stock solutions with the fresh ingredients to a final 2× concentration, filter sterilized, warmed to 37°C, combined with an equal volume of molten autoclave‐sterilized 2× agarose (10 g liter^−1^ at 1×), and poured at 15 ml/plate. Agarose must be used, not agar, which is not sufficiently pure. It is recommended that the LSM‐agarose be kept warm (>55°C) while preparing the plates. Induction of *dacA* deletion was performed on *actA* activating medium, which was made using complete LSM (cLSM) with the addition of 2 mM 2-mercaptoethanol (BME) as previously described (38).

### Mouse infections.

Eight-week-old CD-1 outbred mice (Charles River) were infected intravenously with 1 × 10^5^ CFU in 200 μl of phosphate-buffered saline (PBS). Animals were sacrificed at 48 h, and spleens and livers were harvested in 5 ml or 10 ml in 0.1% IGEPAL CA-630 (Sigma) in water, respectively, and plated for enumeration of bacterial burdens.

### Suppressor generation and identification.

Briefly, mutants were cultured overnight in 5 ml of BHI and genomic DNA was extracted (MasterPure Gram-positive DNA purification kit; Epicentre) according to the manufacturer’s instructions. Genomic DNA (gDNA) was then submitted for library preparation and Illumina sequencing (75PE MiSeq) at the UC Berkeley QB3 Genomics Sequencing Laboratory. Data were assembled and aligned to the 10403S reference genome (GenBank accession no. GCA_000168695.2) demonstrating >50× coverage. Single nucleotide polymorphism (SNP)/indel/structural variations from the wild-type strain were determined (CLC Genomics Workbench; CLC bio).

### (p)ppGpp quantification.

(p)ppGpp was measured as previously described with minor changes (5, 39). Bacteria were grown in low-phosphate *Listeria* synthetic medium (LPLSM) (13). The *dacA^fl^ cre-cbpB* strain and other compared strains required growth in 1% Bacto tryptone to inhibit spontaneous *actA* activation (38). Bacterial overnight cultures were diluted into LPLSM and grown for 2 to 5 h before resuspending 5 × 10^8^ bacteria into 100 μl of either LPLSM or LPLSM plus 1% Bacto tryptone with 20 μCi/ml carrier-free H_3_^32^PO_4_. These cultures were incubated for 120 min at 37°C before resuspending in 50 μl of 13 M formic acid and freeze-thawing 4 times in a dry ice-ethanol bath to lyse the cells. When utilized, serine hydroxamate was added at a concentration of 2 mg/ml for the final 15 min before harvest. Cell debris was removed by centrifugation, and extracts were spotted onto polyethyleneimine (PEI) cellulose thin-layer chromatography (TLC) plates (EMD Millipore) and developed in 1.5 M KH_2_PO_4_, pH 3.4. Dried TLC plates were exposed to phosphor-storage screens (Kodak) for >4 h before imaging on a Typhoon scanner (GE Healthcare). Nucleotides were identified using [γ-^32^P]GTP and E. coli wild-type standard CF1943 (W3110 parental strain), which was generously provided by Michael Cashel (National Institutes of Health). The phosphor-storage screen scan results were quantified using ImageJ software (National Institutes of Health) without background subtraction. The volumes of intensity (without background correction) for identified nucleotide spots were used for calculation of (p)ppGpp levels as follows: (pppGpp + ppGpp)/(pppGpp + ppGpp + GTP).

### Yeast-2-hybrid assay.

The Matchmaker Gold yeast two-hybrid system (Clontech) was used to identify the interaction between CbpB and L. monocytogenes proteins. CbpB was cloned into the pGBKT7 vector (Clontech) by Gibson assembly to generate pGBKT7-CbpB as the bait and then transformed into Y2HGold competent cells. The prey library was constructed from L. monocytogenes genomic DNA and consisted of >100,000 unique 1-kb fragments. Full-length RelA was inserted into the pGADT7 vector (Clontech) by Gibson assembly to generate pGADT7-RelA as the prey to confirm the interaction. Saccharomyces cerevisiae containing pGBKT7-53 and pGADT7-T plasmids was used as a positive control, and S. cerevisiae containing pGBKT7-lam + pGADT7-T plasmids was used as a negative control. Plasmids were transformed into Y2HGold competent cells according to the small-scale transformation and mating procedure described in the Matchmaker Gold yeast two-hybrid system user manual (Clontech). The Y2HGold bait strain and Y187 prey strain were mated in 2× yeast extract-peptone-dextrose agar (YPDA) and plated on an SD/-Leu/-Trp (DDO) or SD/-Ade/-His/-Leu/-Trp/X-a-Gal/aureobasidin A (QDO/X/A) agar plate. The activity of the secreted α-galactosidase enzyme was measured according to manufacturer’s instructions (Clontech). Briefly, diploid S. cerevisiae strains were cultured in DDO medium and supernatants were collected from the overnight culture (20 h postinoculation) and mixed with PNP-α-Gal solution (Clontech). The reaction was terminated after 3 h of incubation, and optical density at 410 nm was measured by SpectraMax (Molecular Devices).

### Protein expression and purification.

The gene encoding the CbpB protein (amino acids 1 to 150) was cloned into a modified pET28a plasmid to generate an N-terminal 6×His-tagged protein. The construct was transformed into E. coli strain BL21 Star (DE3). Cells were grown at 37°C with shaking vigorously at 250 rpm until the optical density at 600 nm (OD_600_) reached 0.8, when they were induced by adding 0.4 mM isopropyl-β-d-1-thiogalactopyranoside (IPTG). Culture was further incubated at 25°C for 12 h and harvested by centrifugation. The cell pellet was resuspended using lysis buffer P500 containing 50 mM phosphate (pH 7.6), 500 mM NaCl, and 20 mM imidazole and lysed by sonication. The lysate was cleared by high-speed centrifugation, and the supernatant was incubated with nickel-nitrilotriacetic acid (Ni-NTA) beads. Beads were washed with lysis buffer, and protein was eluted using lysis buffer supplemented with 250 mM imidazole. The protein was further purified by gel filtration on a HiPrep 16/60 Sephacryl 300 column (GE Healthcare) using a buffer containing 5 mM HEPES (pH 7.6), 200 mM NaCl, and 2 mM dithiothreitol (DTT). The peak fractions containing pure CbpB, which was confirmed by SDS-PAGE, were collected and concentrated to 44 mg/ml. Protein was then flash frozen in liquid nitrogen and stored at −80°C.

### Protein purification for RelA activity experiments.

L. monocytogenes His-tagged CbpB and RelA were transformed into E. coli BL21 and overexpressed at 37°C for 3 h in LB-M9 with addition of 250 μM IPTG. To purify, soluble lysate was run on a HisTrap FF column (GE Healthcare) and eluted by imidazole gradient. Fractions were run on SDS-PAGE to confirm yield and purity and then pooled. Pooled RelA-His and CbpB-His were dialyzed twice into storage buffer (20 mM HEPES, pH 7.8, 200 mM NaCl, 20 mM MgCl_2_, 20 mM KCl, CbpB). Dialyzed proteins were concentrated in Amicon centrifugal filter units (EMD-Millipore) and analyzed by SDS-PAGE, and concentration was determined by Bradford assay (Bio-Rad).

### Enzymatic assays.

pppGpp synthesis assays were performed at 37°C in synthesis buffer (20 mM HEPES, pH 7.8, 200 mM NaCl, 20 mM MgCl_2_, 20 mM KCl) with 1.15 μM RelA-His, 1 mM ATP, 10 μM GTP, and 80 μCi/ml [γ-^32^P]GTP (3,000 mCi/mmol; Perkin Elmer). RelA hydrolase assays were performed at 37°C in hydrolysis buffer (20 mM HEPES, pH 7.8, 200 mM NaCl, 20 mM MgCl_2_, 20 mM KCl, 1 mM MnCl_2_) with 1.15 μM RelA-His, 10 μM pppGpp, and [γ-^32^P]pppGpp. To test effects of CbpB and c-di-AMP, reaction mixtures were supplemented with CbpB-His and/or cyclic-di-AMP at 0 μM, 2.3 μM, or 4.6 μM. Synthetase reactions were run over a course of 8 min, and hydrolase reactions were run over the course of 60 min. Reactions were quenched at time points with 400 mM formic acid on ice. Products were separated by spotting 2 μl of quenched reaction mixture onto PEI cellulose TLC plates (EMD-Millipore) and developing the plates with 1.5 M KH_2_PO_4_, pH 3.4. Phosphorimaging plates (GE Healthcare) were exposed to developed TLC plates and then scanned on a Typhoon FLA9000 scanner (GE Healthcare). Nucleotides were quantified in ImageJ (NIH).

### Protein crystallization and structure determination.

CbpB protein and its mixture with ATP or cyclic-di-AMP were all used for crystallization screening. Free CbpB crystals were grown within 2 days using the sitting-drop vapor-diffusion method at 20°C by mixing 15 mg/ml protein with an equal volume of crystallization buffer containing 100 mM 2-morpholinoethanesulfonic acid (MES) (pH 6.1) and 17% (vol/vol) methyl pentanediol (MPD). Free CbpB structure was also observed using protein supplemented with 4 mM ATP under the same condition. Crystals were cryoprotected using 100% Paratone oil (Hampton Research) and flash frozen in liquid nitrogen. X-ray diffraction data at 1.6-Å resolution were collected at Advanced Light Source (ALS) and processed using HKL2000 (40). The crystal belongs to space group *P*2_1_2_1_2_1_, and each asymmetric unit contains a homodimer.

Crystals of CbpB in complex with cyclic di-AMP were grown using the same method but under a different buffer condition, containing 100 mM imidazole (pH 8.0), 14% (wt/vol) polyethylene glycol (PEG) 8000, and 200 mM CaCl_2_. Crystals were frozen using the same method. Data collection was performed at Advanced Photon Source (APS) and processed using HKL2000. The crystal diffracted to 2.4-Å resolution and belongs to the space group *P*4_3_2_1_2, with each asymmetric unit containing a homodimer.

The structure of free CbpB was solved by the molecular replacement method with Phaser (46) as implemented in PHENIX (41), using the PDB entry 3LQN as the model (60% amino acid identity). The structure was refined with phenix.refine, and manual adjustment was carried out with Coot (42). The structure in complex with cyclic di-AMP was determined by molecular replacement using the structure of free CbpB as the model and refined using phenix.refine. Strong density for the ligand was observed in the electron density map. The crystallographic information is summarized in Table S1 in the supplemental material.

### Citrate quantification.

Bacterial strains were diluted from overnight culture into fresh LSM at an initial OD of approximately 0.1 and cultured to mid-log phase (OD_600_ = 1.0) in LSM (250-ml flask without baffles, 25 ml of medium, 220-rpm shaking, 37°C), and harvested bacteria were washed in PBS, resuspended in an equal volume of citrate assay buffer (see below), and immediately frozen in liquid nitrogen. Bacteria were then thawed and lysed by bead-beating using 0.1-mm zirconia-silica beads (BioSpec) for 15 min. Citrate concentrations were determined using the citrate assay kit (Sigma) according to the manufacturer’s instructions.

### Data availability.

The atomic coordinates and diffraction data for free CbpB and the c-di-AMP complex have been deposited in the Protein Data Bank with entry codes 6XNU and 6XNV.

10.1128/mBio.01625-20.8 TABLE S3 Plasmids used in this study. Download Table S3, TIF file, 2.0 MB. Copyright © 2020 Peterson et al. 2020 Peterson et al. This content is distributed under the terms of the Creative Commons Attribution 4.0 International license.
