# Supplementary figures and images for: (p)ppGpp and c-di-AMP Homeostasis Is Controlled by CbpB in Listeria monocytogenes
Source: mBio. 2020 Aug 25;11(4):e01625-20. doi: 10.1128/mBio.01625-20 (PMC8549634; doi:10.1128/mBio.01625-20)

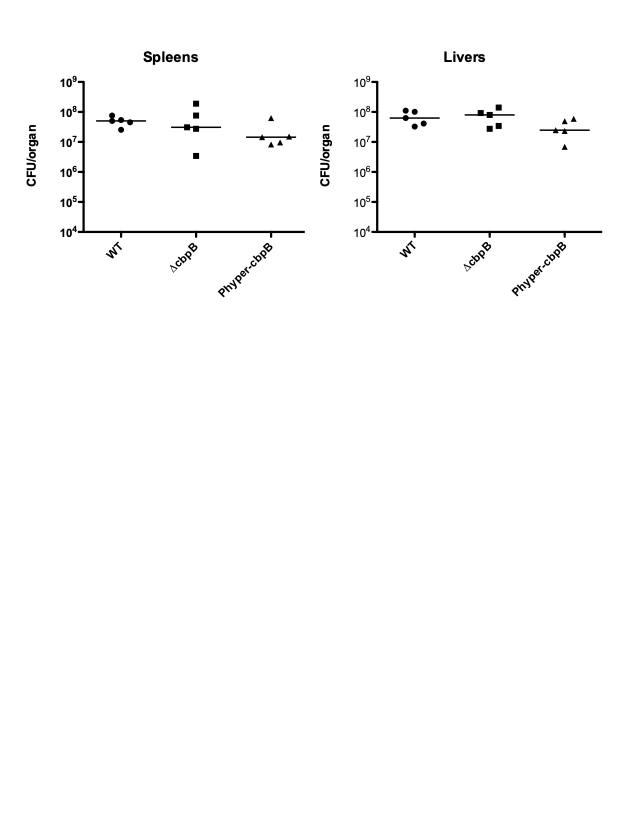

Supplement: FIG S1 [file mbio.01625-20-sf001.tif]

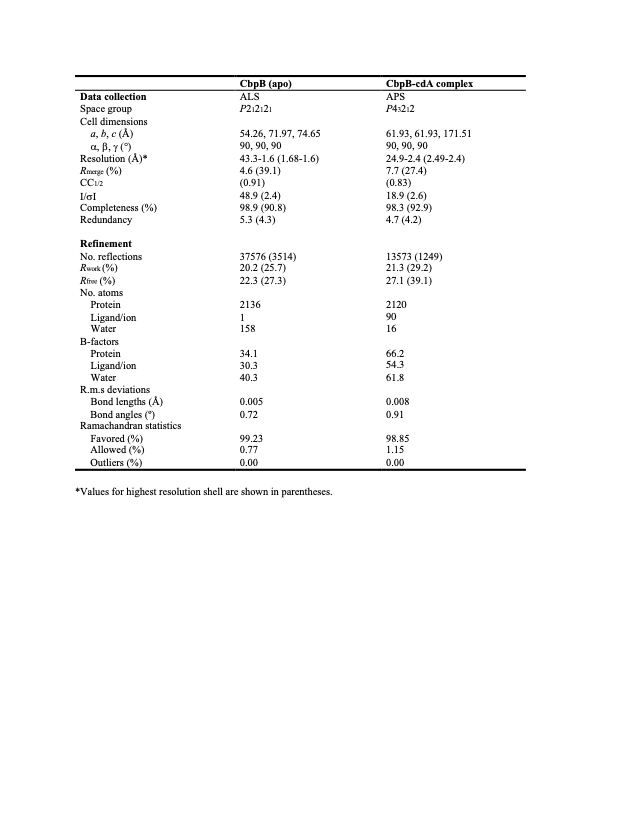

Supplement: TABLE S1 [file mbio.01625-20-st001.tif]

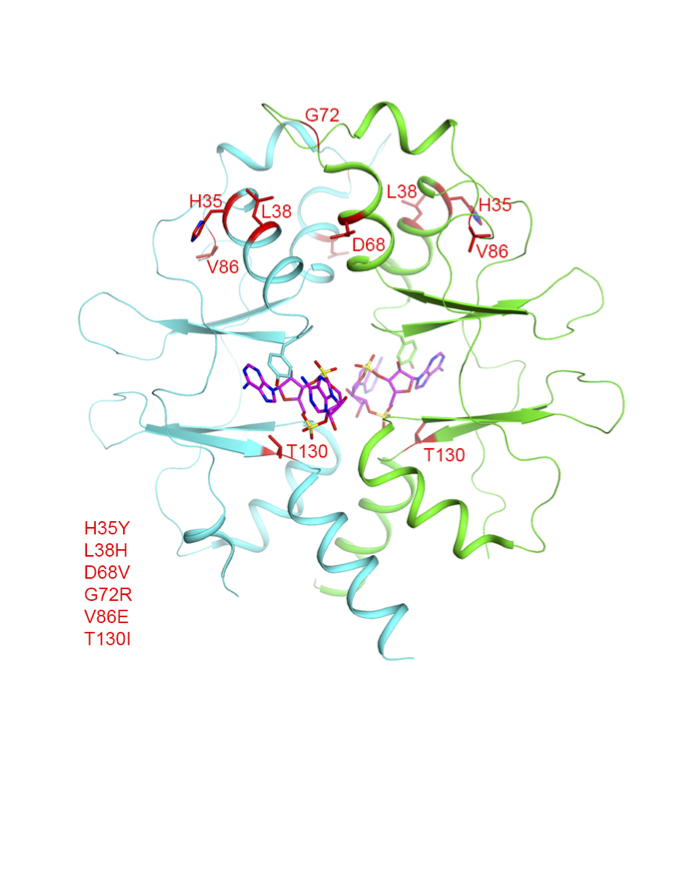

Supplement: FIG S2 [file mbio.01625-20-sf002.tif]

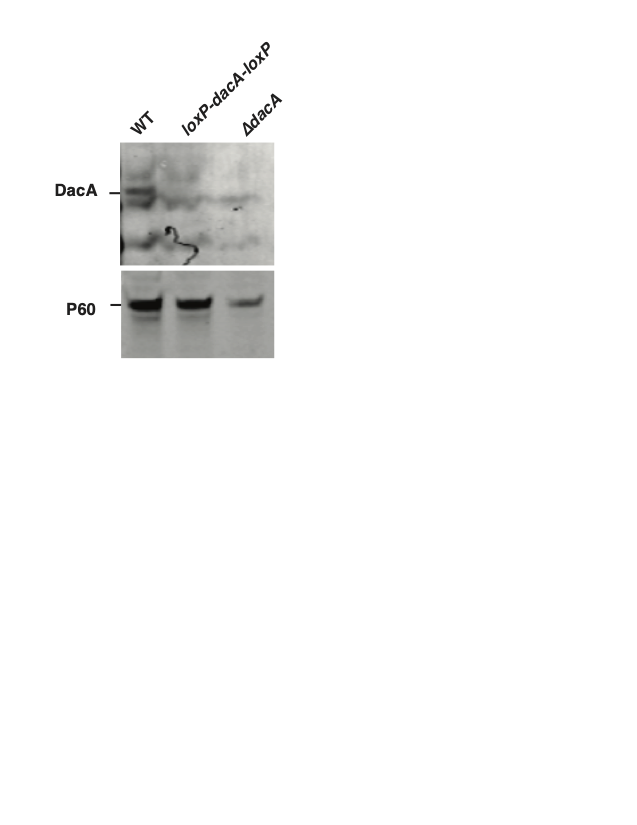

Supplement: FIG S3 [file mbio.01625-20-sf003.tif]

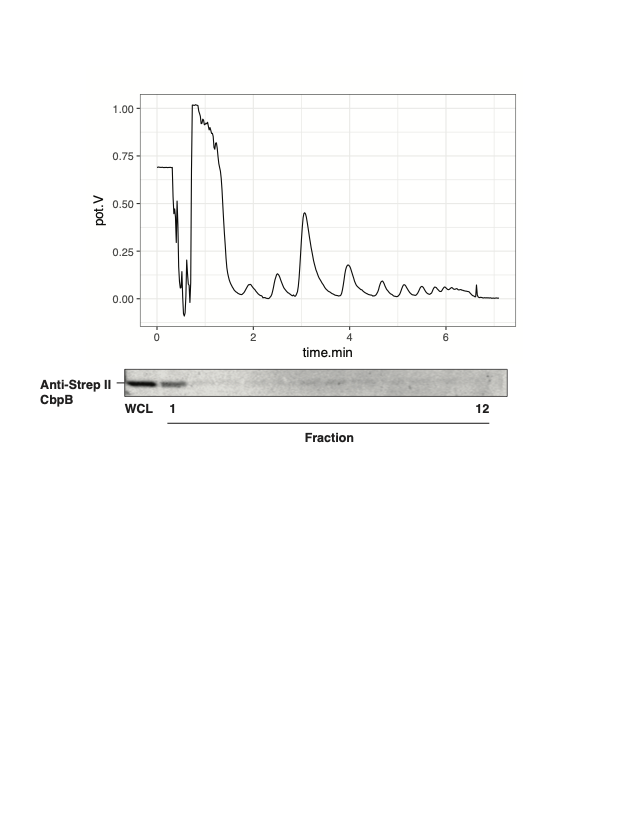

Supplement: FIG S4 [file mbio.01625-20-sf004.tif]

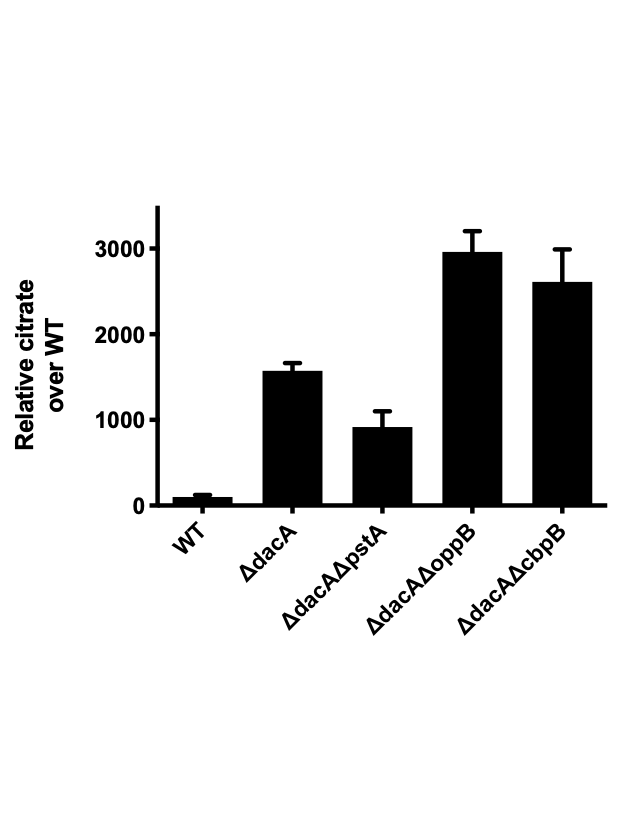

Supplement: FIG S5 [file mbio.01625-20-sf005.tif]

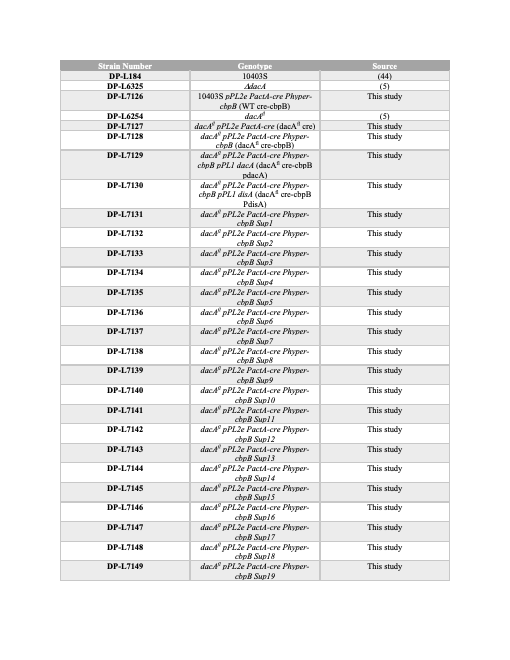

Supplement: TABLE S2 [file mbio.01625-20-st002.tif]

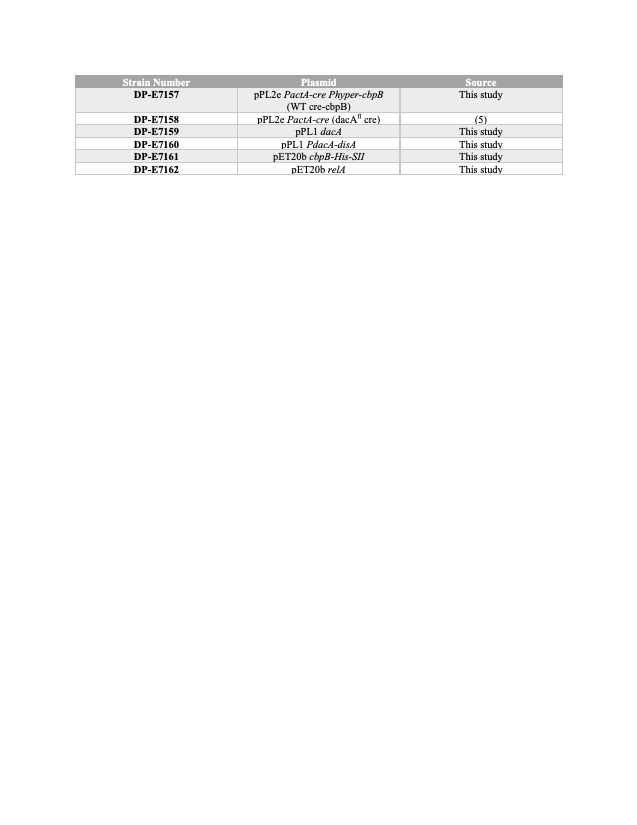

Supplement: TABLE S3 [file mbio.01625-20-st003.tif]
